# Acceptance and Preference for COVID-19 Vaccine among Japanese Residents at Early Stage of the Epidemic in Japan

**DOI:** 10.3390/vaccines11010157

**Published:** 2023-01-11

**Authors:** Jiwen Wu, Guoxi Cai, Yi Fan, Kazuhiko Arima, Yulan Lin, Liping Wong, Zhuo Zhang, Taro Yamamoto, Kouichi Morita, Akira Yoshikawa, Yixiao Lu, Kiyoshi Aoyagi

**Affiliations:** 1 Department of Public Health, Nagasaki University Graduate School of Biomedical Sciences, Nagasaki 852-8523, Japan; 2 Department of Human Anatomy, School of Basic Medical Sciences, Fujian Medical University, Fuzhou 350108, China; 3 Department of International Health and Medical Anthropology, Institute of Tropical Medicine (NEKKEN), Nagasaki University, Nagasaki 852-8523, Japan; 4 Nagasaki Prefectural Institute of Environment and Public Health, Nagasaki 856-0026, Japan; 5 Department of Epidemiology and Health Statistics, Fujian Provincial Key Laboratory of Environment Factors and Cancer, School of Public Health, Fujian Medical University, Fuzhou 350108, China; 6 Centre for Epidemiology and Evidence-Based Practice, Department of Social and Preventive Medicine, Faculty of Medicine, University Malaya, Kuala Lumpur 50603, Malaysia; 7 Department of Virology, Institute of Tropical Medicine (NEKKEN), Nagasaki University, Nagasaki 852-8523, Japan; 8 Dejima Infectious Disease Research Alliance, Nagasaki University, Nagasaki 852-8523, Japan

**Keywords:** COVID-19 vaccine, attitude, vaccination acceptance, vaccine choice

## Abstract

Background: This study aimed to survey the attitudes toward COVID-19 vaccines and their acceptability among the Japanese public as soon as the United States Food and Drug Administration (FDA) authorized vaccines and their rollouts started around the world. Methods: An anonymous cross-sectional survey was conducted in Japan between 4 January and 5 March 2021. A questionnaire was administered to evaluate attitudes toward COVID-19 vaccines according to demographic characteristics, vaccine characteristics, and vaccine production. Results: A total of 1037 completed responses were received. More than half (63.5%) of the participants responded positively (extremely likely/likely) toward COVID-19 vaccines. The highest acceptance to be vaccinated was discovered among the youngest age group. As expected, participants who had never delayed acceptance or refused the vaccine in their history of vaccination had a significantly higher willingness to be vaccinated against COVID-19 (*p* < 0.001). Females (OR = 2.66, 95% CI: 1.99–3.58) and participants who had ever delayed acceptance or refuse the vaccine (OR = 3.49, 95% CI: 2.42–5.05) had higher odds of COVID-19 vaccine hesitancy. Participants with a postgraduate degree (OR = 0.64, 95% CI: 0.40–1.00) presented the highest willingness to be vaccinated against COVID-19. More than two-thirds (72.9%, 95% CI: 70.4%–75.8%) of the participants did not mind a booster dose required following primary vaccination. A total of 63.2% (95% CI: 60.0%–66.0%) of the participants only accepted a nearly 90% effective or above vaccine at preventing COVID-19. At the same, 86.4% (95% CI: 84.4%–88.4%) of the participants reported only accepting a vaccine with minor side effects. Conclusions: The moderate levels of COVID-19 vaccine acceptance found in the early phase of the pandemic demonstrate that it is important to improve the implementation of effective management for vaccine promotion and the acceptability of the vaccine to slow or delay transmission.

## 1. Introduction

The enormous burdens of morbidity and mortality, including economic, social, and mental impacts due to coronavirus disease 2019 (COVID-19), have affected people worldwide since the World Health Organization (WHO) declared a global pandemic on March 11, 2020 [1,2]. Over 2020–2021, 5.42 million deaths related to COVID-19 have been reported [3]. Vaccines are a useful and powerful weapon for pandemic control when a safe and effective one becomes available. To achieve herd immunity and to prevent community transmission (including the emergence of highly contagious variants such as the delta and omicron variants) [4], the acceptance of COVID-19 vaccines must be improved. The COVID-19 vaccination campaign began in Japan in February 2021 with priority vaccinations for healthcare workers and the elderly, followed by other populations, sequentially. In addition to presenting substantial obstacles in the public health arena, vaccine hesitancy also contributes to inadequate COVID-19 vaccine coverage, increasing the risk of infection among those who remain susceptible in the community [5,6]. Therefore, it is necessary to understand the acceptability of vaccines against COVID-19 among the general public in Japan during the early phase of the pandemic when COVID-19 vaccines were just available.

Vaccine hesitancy, as has been confirmed by multiple studies, is a threat to preventing persistent community transmission and controlling COVID-19, and, in fact, WHO named it one of the top ten threats to global health in 2019 [7,8,9]. COVID-19 vaccine hesitancy is not yet understood with regard to the availability of various vaccines with different characteristics, such as effectiveness against virus variants, adverse side effects, administration doses, manufacturing platforms, and durations of protection, when the vaccines were first approved for use [10,11]. Demographical factors, such as age, gender, educational level, and economic factors, were associated with vaccine hesitancy [12]; meanwhile, misinformation and religious, cultural, or erroneous knowledge on immunization or immunization services are also associated with an unwillingness to receive COVID-19 vaccines [13]. However, skepticism regarding vaccine efficacy and unfavorable announcements of adverse side effects could prevent and delay vaccination intention, which may lead to an extremely low vaccination rate, influencing the indirect protection for the overall community [14,15]. Vaccine efficacies ranging from 50 to 95% have been reported [16]. COVID-19 vaccines being developed using messenger RNA (mRNA) technology and produced by different manufacturers around the world may also be associated with hesitancy. In order to improve vaccine uptake and coverage, it is crucial to understand the characteristics of vaccines that influence the acceptance and choice of a vaccine.

In the early phase of the COVID-19 pandemic, a heterogeneous willingness to accept vaccines was found; participants from Asian nations and middle-income countries had relatively high acceptance [16]. A multi-country survey was conducted to assess COVID-19 vaccination acceptance as soon as the United States Food and Drug Administration (FDA) authorized vaccines and their roll-out started around the world [17]. Japan was also one of the participatory countries in the multi-country survey.

This study synthesizes the findings on the acceptance of and attitudes toward COVID-19 vaccine characteristics and also explores the factors influencing COVID-19 vaccine choice from the data collected in Japan. These findings provide insights into the challenges surrounding the introduction of new vaccines and will be useful for future infectious disease pandemics. Furthermore, government agencies can use this knowledge to devise strategies to improve the vaccination rate in the future.

## 2. Materials and Methods

### 2.1. Study Design and Participants

We conducted a cross-sectional web-based survey using an online questionnaire between 4 January and 5 March 2021. A total of 1037 individuals from Japan participated in this survey. It was necessary for individuals to be 18 years or older, to be Japanese residents who had not yet received a COVID-19 vaccination, and to provide informed consent online.

### 2.2. Study Instrument

The questionnaire was developed in English. A native language option for the questions was available for surveys carried out in Japan. The items of the questions were content validated by content experts. Translations into target languages were carried out using standard forward–backward translation by native speakers. The translated questionnaires were also validated by new independent bilingual native speakers (Supplementary File). To ensure valid and reliable responses, we carried out survey data cleaning before analyses. Duplicate responses were removed. The survey had three sections: (1) general information, (2) attitudes towards COVID-19 vaccine characteristics and factors influencing vaccine choice, and (3) acceptability of COVID-19 vaccines.

The general information of the questionnaire: (1) age, sex, and highest education attainment; (2) if they had ever delayed acceptance of or refused a vaccine despite the availability of a vaccine service. The response options were “yes” or “no”.

The attitudes towards COVID-19 vaccines were examined by asking participants to state their opinions on 6 statements, including number of doses, effectiveness threshold, side effects, duration of protection, technology used in COVID-19 vaccine production, and producing country.

The acceptability of COVID-19 vaccines was assessed with the response options “extremely likely”, “likely”, “unlikely”, and “extremely unlikely”.

### 2.3. Statistical Analysis

R software (version 4.1.3) was used to analyze the data. The distributions of socio-demographics across vaccine willingness were examined using a χ2 test or Fisher’s exact test, if appropriate. The effect directions of demographic characteristics on vaccine characteristics influencing vaccination attitudes were first evaluated with the Spearman coefficient and visualized with a heatmap. Then, a univariate logistic regression followed by a multivariate logistic regression were applied to explore the effect sizes of the demographic characteristics on attitudes towards COVID-19 vaccines or vaccine characteristics influencing vaccination attitudes. A result with a *p*-value < 0.05 was considered statistically significant.

## 3. Results

In total, 1037 participants across Japan were admitted to this study. As shown in Table 1, 56.0% of the study participants were female and the distribution of age was nearly even in each group. More than half of the participants (57.9%) had a bachelor’s degree and above and most participants (84.5%) had not ever delayed acceptance of or refused a vaccine despite the availability of vaccine services.

### 3.1. COVID-19 Vaccine Acceptance in Japan’s Participants

**Figure 1:**
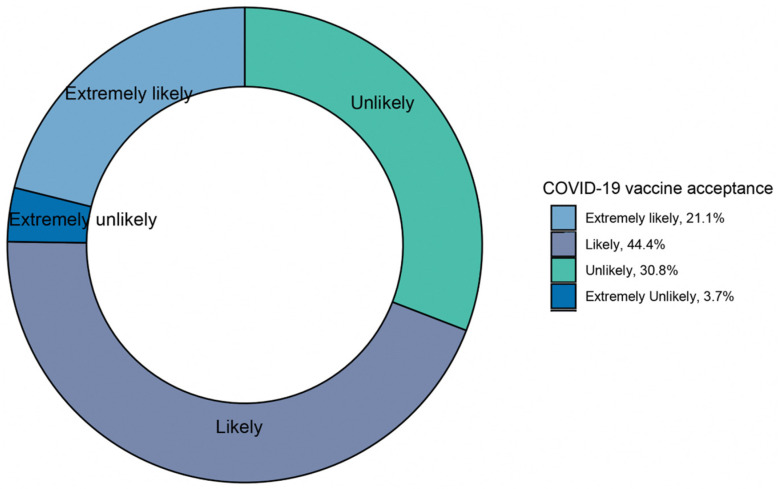
COVID-19 vaccine acceptance of the participants in Japan (*N* = 1037).

The proportion of responses for COVID-19 vaccine acceptance is shown in Figure 1. In the main, 63.5% of the participants responded positively (extremely likely/likely) toward COVID-19 vaccines, whereas 34.5% (extremely unlikely/unlikely) responded negatively to COVID-19 vaccine intent. With a more specific breakdown, 21.1% (95% CI: 18.6%–23.5%) of the participants were extremely likely to accept a COVID-19 vaccine, followed by 44.4% (95% CI: 41.5%–47.4%) of the participants who responded likely if it is recommended by the government. Only a small proportion reported extremely unlikely to accept a COVID-19 vaccine (3.7%, 95% CI: 2.6%–4.9%).

### 3.2. Effect of Demographic Characteristics on the Attitudes towards COVID-19 Vaccines

The willingness to be vaccinated against COVID-19 by the demographics of the participants across Japan is shown in Table 2. The highest willingness to be vaccinated was discovered among the youngest age group, while the greatest amount of uncertainty was discovered among 40–49-year-olds and 30–39-year-olds. A significantly higher proportion of males were extremely likely/likely to accept a COVID-19 vaccine than females (*p* = 0.007). As expected, participants who had never delayed acceptance of or refused a vaccine had a significantly higher willingness to be vaccinated against COVID-19 (*p* < 0.001). The distribution of the willingness to be vaccinated against COVID-19 among the participants with the highest educational attainment was not statistically significant (*p* = 0.053), while a higher willingness to be vaccinated against COVID-19 was found in participants with high educational attainment.

The factors influencing COVID-19 vaccine willingness with a logistic regression model are shown in Table 3. Females (OR = 2.66, 95% CI: 1.99–3.58) had higher odds of a positive attitude than male counterparts.

Participants who had ever delayed acceptance of or refused a vaccine (OR = 3.49, 95% CI: 2.42–5.05) had higher odds of COVID-19 vaccine hesitancy after adjusting for age, gender, highest educational attainment, and ever delayed acceptance of or refused a vaccine. Participants who had a postgraduate degree (OR = 0.64, 95% CI: 0.40–100) presented the highest willingness to be vaccinated against COVID-19 compared with participants in secondary school and with lower degree. Compared with the youngest age group, a gradual increase in COVID-19 vaccine hesitancy with age was found; however, the point estimation revealed that the oldest age group presented a positive willingness to be vaccinated against COVID-19.

We further explore the associations between influencing factors and the willingness to be vaccinated against COVID-19 among participants who had not ever delayed acceptance of or refused a vaccine. Supplementary Table S1 presents the results, which remained substantially the same; males and participants who had a high education attainment showed a higher willingness to be vaccinated against COVID-19.

### 3.3. Effects of Demographic Characteristics on the Vaccine Choice

**Figure 2:**
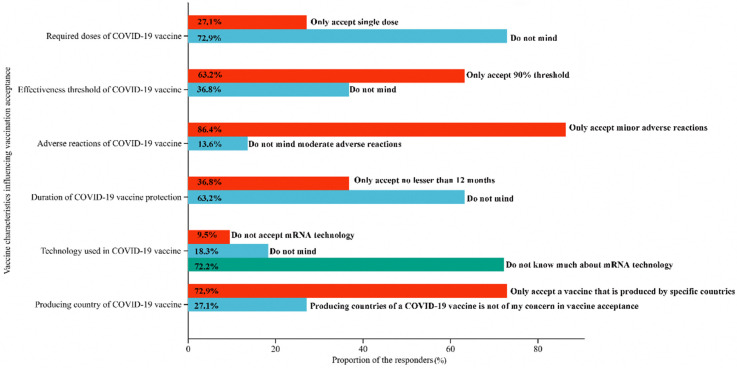
The choice of vaccine characteristics influencing vaccination acceptance (*N* = 1037).

The results on attitudes toward vaccine characteristics influencing vaccination attitudes are shown in Figure 2. More than two-thirds (72.9%, 95% CI: 70.4%–75.8%) of the participants did not mind a booster dose required following primary vaccination. A total of 63.2% (95% CI: 60.0%–66.0%) of the participants only accept nearly 90% effective or above vaccines for preventing COVID-19. In the same, 86.4% (95% CI: 84.4%–88.4%) of the participants reported only accepting a vaccine that has minor side effects. For the duration of protection of a COVID-19 vaccine, over half (63.2%, 95% CI: 60.3%–66.1%) of the participants did not mind a duration of protection of a vaccine between 6 to 12 months. On the matter of the technology used in COVID-19 vaccine production, only 9.5% (95% CI: 7.6%–11.3%) of the participants did not accept mRNA technology in COVID-19 vaccine production, while the majority (72.2%, 95% CI: 69.5%–74.9%) of the participants did not know much about mRNA technology. Over two-thirds (72.9%, 95% CI: 70.3%–75.7%) of the participants only accepted a vaccine that is produced by specific countries.

**Figure 3:**
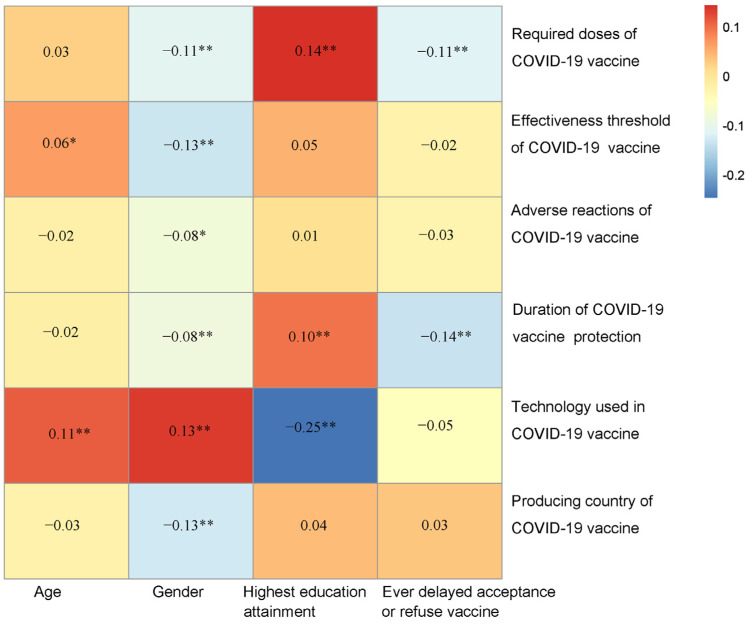
The correlations between demographic characteristics and vaccine characteristics influencing vaccination attitudes of all participants in Japan. * *p* < 0.05, ** *p* < 0.01.

**Figure 4:**
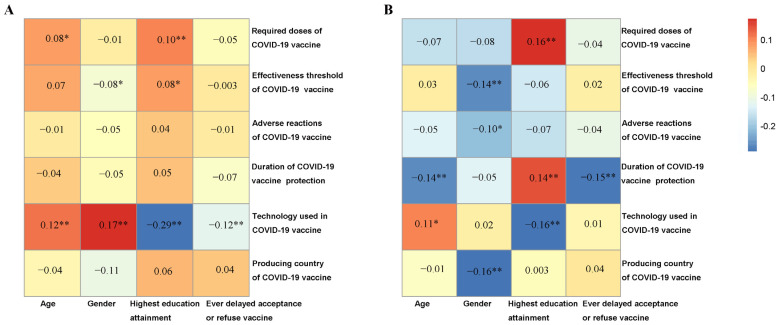
The correlations between demographic characteristics and vaccine characteristics influencing vaccination attitudes of participants in Japan. (**A**) In extremely likely/likely participants and (**B**) in extremely unlikely/unlikely participants. * *p* < 0.05, ** *p* < 0.01.

Next, the correlation between demographic characteristics and vaccine characteristics influencing attitudes towards vaccines was visualized with a heatmap (Figure 3). Among all the participants, females reported lower knowledge of mRNA technology and showed little concern for the effectiveness threshold of a COVID-19 vaccine. In addition, participants with higher education attainment presented little concern for a booster dose required following primary vaccination and had more knowledge of mRNA technology. We further explored the associations between demographic characteristics and vaccine characteristics influencing vaccination attitudes among participants who were extremely likely/likely to be vaccinated and participants who were extremely unlikely/unlikely to be vaccinated separately (Figure 4). A similar relationship between demographic characteristics and vaccine characteristics influencing vaccination attitudes was observed between all participants and participants who were extremely likely/likely to be vaccinated. However, among participants who were extremely unlikely/unlikely to be vaccinated, females showed more attentiveness to the doses, effectiveness, side effects, and duration of protection of a COVID-19 vaccine. Moreover, participants who had high education attainment showed more attentiveness to the effectiveness and side effects of a COVID-19 vaccine.

A multivariate logistic regression was also performed to examine the relationship between demographic characteristics and vaccine characteristics influencing vaccination attitudes. As shown in Table 4, participants with a bachelor’s degree and above may have been more likely to accept only one dose of a vaccine and a vaccine with a duration protection of no shorter than 12 months. Compared with the youngest age group, participants aged above 40 years old expressed a higher likelihood to accept a vaccine with nearly 90% effectiveness in preventing COVID-19, and those aged above 50 had less knowledge of the technology used in COVID-19 vaccines. As for gender, females showed little concern for vaccine characteristics influencing their vaccination attitudes, which is similar to the results of the heatmap. The same analyses were performed among participants who were extremely likely/likely to be vaccinated, and a similar effect is observed in Supplementary Table S2.

## 4. Discussion

This investigation aimed to assess attitudes around COVID-19 acceptance among the general public in Japan during the early phase of the pandemic, and the findings could offer insight into the strategies required to effectively address issues surrounding vaccine hesitancy in pandemics of infectious diseases.

Compared with a previous global study of COVID-19 vaccination among people in Japan [15], our study confirmed the relatively low intention for COVID-19 vaccination during the early phase of the pandemic. In general, the majority in Asian nations had high vaccination acceptance intentions, wherein the public have strong institutional trust as well as historical and socio-cultural factors, but a notable exception was Japan [16]. Our findings showed relatively low intentions for COVID-19 vaccination (63.5%) among people in Japan during the early phase of the pandemic when COVID-19 vaccines were available, and this was similar to the results found of a vaccination rate of only 65.7% in a study of COVID-19 vaccination intention conducted around the same time as our study [18].

After adjusting for age, gender, highest educational attainment, and ever delayed acceptance of or refused of a vaccine, females and participants who had ever delayed acceptance of or refused a vaccine experienced higher levels of COVID-19 vaccine hesitancy. In this study, higher levels of COVID-19 vaccine hesitancy were seen in these groups due to pre-existing vaccine hesitancy, which is based on a lower trust in healthcare professionals, the health system, and the government [19,20]. In particular, there is a notorious refusal rate in Japan for the human papillomavirus (HPV) vaccination for teenage girls. A combination of negative perceptions and historical experiences has caused a sharp decline in the HPV vaccination rate among the younger generation in Japan. Compared with the youngest age group, a gradual increase in COVID-19 vaccine hesitancy with age was found; however, the point estimation revealed that the oldest age group presented a positive willingness to be vaccinated against COVID-19. Many countries are currently administering COVID-19 vaccination, and older people are considered the first priority group for a COVID-19 vaccination program. Therefore, providing older adults with information and support is critical to enhancing vaccination coverage. Research shows that better-educated individuals are more likely to understand public health messages and access reliable information on the safety and effectiveness of vaccines [21]. An increasing trend of willingness to be vaccinated against COVID-19 was found in those with educational attainments, and participants who had a postgraduate degree presented the highest willingness to be vaccinated against COVID-19 compared with participants in secondary school and lower degrees in our study. Individuals who have the highest education attainment have a better opportunity to be educated and also have better access to information, especially through healthcare professionals. Perhaps this also explains why participants who had a postgraduate degree in this study had high knowledge and undertook necessary precautions to prevent the development of community transmission, such as pursuing a healthy lifestyle and obtaining more information on immunization. In many studies, high educational levels have been reported to be associated with better knowledge [22,23,24].

Vaccine choice factors revealed that most participants would only accept a vaccine with minor side effects, indicating that the safety of the newly developed COVID-19 vaccines was extremely important. It is imperative for the public to know that the COVID-19 vaccines have been rigorously tested, including through large clinical trials, despite being rolled out for emergency use, and that early trial data indicated that severe adverse effects were extremely rare [25,26] and the vaccines have been found to be safe and effective. Regarding the technology used in COVID-19 vaccine production, the majority of participants did not know much about mRNA technology, especially those over 50 who had less knowledge of the technology used in COVID-19 vaccines. In spite of the new biotechnology behind new mRNA vaccines, communicating their safety and efficacy is still a challenge [27,28]. In order to eliminate skepticism among reluctant respondents, it is critical to ensure transparency and timely and accurate information about COVID-19 vaccines in terms of their efficacy and side effects. Of notable importance, this study found that 63.2% of the participants would only accept a nearly 90% effective or above vaccine for preventing COVID-19. A lower level of effectiveness may lead people to be less willing to accept a vaccine, believing that a lower level of effectiveness indicates inferiority.

Study limitations must be considered when interpreting the results. Firstly, an online survey may lead to sampling bias, so the results may not be generalizable to the larger community, as indicated by the absence of a representative from some of the prefectures. Secondly, this study was a cross-sectional study, although, we were able to identify associations between attitudes towards COVID-19 vaccine characteristics and acceptability; however, we could not infer cause and effect. Lastly, as the responses were self-reported, there may have been self-reporting bias and a tendency to report responses that are socially desirable. Although the current study has limitations, it is the first survey of its kind to be conducted in Japan, providing valuable first-hand information about the attitudes and acceptance of COVID-19 vaccines in Japan.

## 5. Conclusions

The current study showed that Japan’s public had a moderate acceptance of COVID-19 vaccines when the vaccines were newly introduced. High vaccination coverage rates indirectly protect the overall community by slowing transmission. Clearly, the different types of COVID-19 vaccines with diverse characteristics currently available may increase uncertainty and difficulty in making a decision, resulting in people delaying or refusing vaccination. The present study underlines the importance of herd immunity during early epidemic outbreaks, targeted at the hesitancy segment of the population, to moderate the perceived risks of COVID-19 vaccination and generate long-term benefits from the immunity group. However, this strategy usually needs strong and sustained support from the government.

## Figures and Tables

**Table 1:** The descriptive demographics of the participants in Japan (*N* = 1037).

Socio-Demographic	*N*	
Age, years		
18–29	182	17.6%
30–39	200	19.3%
40–49	207	20.0%
50–59	167	16.1%
60–69	281	27.1%
Gender		
Male	456	44.0%
Female	581	56.0%
Highest education level		
Secondary school and below	237	22.9%
Certificate/A-level/diploma	199	19.2%
Bachelor’s degree	417	40.2%
Postgraduate degree	184	17.7%
Ever delayed acceptance of or refused vaccine despite availability of vaccine service		
No	876	84.5%
Yes	161	15.5%

**Table 2:** Willingness to be vaccinated against COVID-19 by demographics of the participants in Japan (*N* = 219).

Demographic	Extremely Likely	Likely	Unlikely	Extremely Unlikely	*p*
Age, years					0.007 *
18–29	48 (26.4%)	83 (45.6%)	44 (24.2%)	7 (3.8%)	
30–39	34 (17.0%)	89 (44.5%)	67 (33.5%)	10 (5.0%)	
40–49	29 (14.0%)	87 (42.0%)	80 (38.6%)	11 (5.3%)	
50–59	35 (21.0%)	72 (43.1%)	55 (32.9%)	5 (3.0%)	
60 and above	73 (26.0%)	129 (45.9%)	74 (26.3%)	5 (1.8%)	
Gender					<0.001
Male	124 (27.2%)	225 (49.3%)	82 (18.0%)	25 (5.5%)	
Female	95 (16.4%)	235 (40.4%)	238 (41.0%)	13 (2.2%)	
Highest education level					0.053
Secondary school and below	38 (16.0%)	104 (43.9%)	81 (34.2%)	14 (5.9%)	
Certificate/A-level/diploma	35 (17.6%)	85 (42.7%)	71 (35.7%)	8 (4.0%)	
Bachelor’s degree	99 (23.7%)	191 (45.8%)	117 (28.1%)	10 (2.4%)	
Postgraduate degree	47 (25.5%)	80 (43.5%)	51 (27.7%)	6 (3.3%)	
Ever delayed acceptance of or refuse vaccine despite availability of vaccine service					<0.001
No	201 (22.9%)	407 (46.5%)	243 (27.7%)	25 (2.9%)	
Yes	18 (11.2%)	53 (32.9%)	77 (47.8%)	13 (8.1%)	

* Fisher’s exact test.

**Table 3:** Demographic characteristics influencing COVID-19 vaccination acceptance of the participants in Japan (*N* = 1037).

Demographic	Participants (*N* = 1037)	Extremely Unlikely/Unlikely vs. Extremely Likely/Likely to Accept COVID-19 Vaccination	
		Univariate OR (95% CI)	Multivariate OR (95% CI)
Age, years			
18–29	182	1.00 (reference)	1.00 (reference)
30–39	200	1.61 (1.04, 2.48) *	1.63 (1.04, 2.58) *
40–49	207	2.01 (1.32, 3.09) *	1.88 (1.20, 2.58) *
50–59	167	1.44 (0.91, 2.26)	1.25 (0.77, 2.03)
60 and above	281	1.00 (0.66, 1.52)	0.80 (0.51, 1.26)
Gender			
Male	456	1.00 (reference)	1.00 (reference)
Female	581	2.48 (1.89, 3.26) *	2.66 (1.99, 3.58) *
Highest education attainment			
Secondary school and below	237	1.00 (reference)	1.00 (reference)
Certificate/A-level/diploma	199	0.98 (0.66, 1.44)	0.82 (0.53, 1.23)
Bachelor’s degree	417	0.65 (0.46, 0.91) *	0.73 (0.51, 1.05)
Postgraduate degree	184	0.67 (0.44, 1.00)	0.64 (0.40, 1.00)
Ever delayed acceptance of or refuse vaccine despite availability of vaccine service			
No	876	1.00 (reference)	1.00 (reference)
Yes	161	2.87 (2.04, 4.06) *	3.49 (2.42, 5.05) *

* *p* < 0.05.

**Table 4:** Vaccine characteristics influencing vaccination acceptance by demographics of the participants in Japan.

Demographic	Required Doses of COVID-19 Vaccine ^a^	Effectiveness Threshold of COVID-19 Vaccine ^b^	Adverse Reactions of COVID-19 Vaccine ^c^	Duration of COVID-19 Vaccine Protection ^d^	Technology Used in COVID-19 Vaccine ^e^	Producing Country of COVID-19 Vaccine ^f^
	Multivariate OR (95% CI)	Multivariate OR (95% CI)	Multivariate OR (95% CI)	Multivariate OR (95% CI)	Multivariate OR (95% CI)	Multivariate OR (95% CI)
Age, years						
18–29	1.00 (reference)	1.00 (reference)	1.00 (reference)	1.00 (reference)	1.00 (reference)	1.00 (reference)
30–39	1.14 (0.72, 1.82)	0.83 (0.53, 1.28)	0.98 (0.55, 1.71)	1.03 (0.67, 1.58)	0.69 (0.36, 1.29)	1.25 (0.80, 1.95)
40–49	0.81 (0.50, 1.30)	0.58 (0.37, 0.89) *	1.31 (0.72, 2.39)	0.86 (0.56, 1.33)	0.81 (0.43, 1.50)	1.52 (0.96, 2.41)
50–59	0.80 (0.48, 1.31)	0.46 (0.29, 0.73) *	1.43 (0.75, 2.78)	0.77 (0.48, 1.22)	0.40 (0.17, 0.87) *	1.38 (0.85, 2.26)
60 and above	0.64 (0.40, 1.00)	0.62 (0.41, 0.94) *	1.08 (0.62, 1.85)	0.97 (0.65, 1.46)	0.44 (0.22, 0.86) *	1.13 (0.74, 1.73)
Gender						
Male	1.00 (reference)	1.00 (reference)	1.00 (reference)	1.00 (reference)	1.00 (reference)	1.00 (reference)
Female	1.56 (1.15, 2.10) *	1.61 (1.23, 2.10) *	1.54 (1.06, 2.23) *	1.42 (1.08, 1.86) *	1.04 (0.67, 1.63)	1.68 (1.26, 2.24) *
Highest education level						
Secondary school and below	1.00 (reference)	1.00 (reference)	1.00 (reference)	1.00 (reference)	1.00 (reference)	1.00 (reference)
Certificate/A-level/diploma	0.87 (0.57, 1.31)	1.43 (0.94, 2.18)	1.20 (0.66, 2.21)	0.78 (0.52, 1.16)	0.99 (0.47, 2.03)	1.49 (0.94, 2.38)
Bachelor’s degree	0.64 (0.44, 0.91) *	0.80 (0.57, 1.13)	0.92 (0.57, 1.47)	0.66 (0.47, 0.93) *	0.85 (0.46, 1.60)	1.14 (0.79, 1.65)
Postgraduate degree	0.31 (0.18, 0.51) *	0.89 (0.58, 1.36)	1.27 (0.70, 2.36)	0.60 (0.39, 0.91) *	2.19 (1.17, 4.17) *	0.92 (0.59, 1.43)
Ever delayed acceptance of or refused vaccine despite availability of vaccine service						
No	1.00 (reference)	1.00 (reference)	1.00 (reference)	1.00 (reference)	1.00 (reference)	1.00 (reference)
Yes	2.06 (1.42, 2.96) *	1.23 (0.86, 1.78)	1.33 (0.80, 2.34)	2.17 (1.54, 3.07)	2.11 (1.25, 3.45)	0.87 (0.60, 1.27)

^a^: only accept single dose vs. do not mind, ^b^: only accept 90% threshold vs. do not mind, ^c^: only accept minor adverse reactions vs. do not mind moderate adverse reactions, ^d^: only accept lesser than 12 months vs. do not mind moderate adverse reactions, ^e^: do not accept mRNA technology vs. do not know much about mRNA technology/do not mind, and ^f^: only accept a vaccine that is produced by specific countries vs. producing countries of a COVID-19 vaccine is not of my concern in vaccine choice. * *p* < 0.05.

## Data Availability

The raw data supporting the conclusions of this article will be made available by the authors, without undue reservations, to any qualified researchers.

## Supplementary Materials

The following supporting information can be downloaded at https://www.mdpi.com/article/10.3390/vaccines11010157/s1. Table S1: The associations between influencing factors and the willingness to be vaccinated against COVID-19 among participants who have never delayed acceptance or refused vaccination (*N* = 876); Table S2: Vaccine characteristics influencing vaccination acceptance by demographics of the participants who are extremely likely/likely to be vaccinated against COVID-19 (*N* = 679).
