# Supplementary material for: Acceptance and Preference for COVID-19 Vaccine among Japanese Residents at Early Stage of the Epidemic in Japan
Source: Vaccines (Basel). 2023 Jan 11;11(1):157. doi: 10.3390/vaccines11010157 (PMC9861095; doi:10.3390/vaccines11010157)
Supplement: Supplementary file 1 [file vaccines-11-00157-s001.zip › vaccines-2103941-supplementary.pdf]

Table S1 The associations between influencing factors and the willingness to be vaccinated against COVID-19 among participants who have never delayed acceptance or refused vaccination (*N*=876).

Demographic	Participants (*N*=1037)	Extremely unlikely/unlikely vs extremely likely/likely to accept COVID-19 vaccination	
		Univariate OR (95%CI)	Multivariate OR (95%CI)
Age			
18-29	160	1.00(Reference)	1.00(Reference)
30-39	170	1.40(0.87,2.24)	1.67(1.07,2.61)
40-49	179	1.73(1.09,2.75)	1.88(1.21,2.93)
50-59	139	1.22(0.74,2.01)	1.33(0.83,2.14)
60 and above	228	0.74(0.46,1.18)	0.89(0.57,1.39)
Gender			
Male	375	1.00(Reference)	1.00(Reference)
Female	501	2.51(1.84,3.43)	2.40(1.81,3.19)
Highest education attainment			
Secondary school and below	193	1.00(Reference)	1.00(Reference)
Certificate/A-Level/Diploma	172	0.98(0.63,1.51)	0.78(0.52,1.17)
Bachelor degree	359	0.75(0.51,1.10)	0.70(0.49,1.00)
Postgraduate degree	152	0.76(0.47,1.20)	0.66(0.42,1.01)

Table S2 Vaccine characteristics influencing vaccination acceptance by demographics of the participants who are extremely likely/likely to be vaccinated against COVID-19 (*N*=679)

Demographic	Required doses of COVID-19 vaccine[a]	Effectiveness threshold of COVID-19 vaccine[b]	Adverse reactions of COVID-19 vaccine[c]	Duration of COVID-19 vaccine protection[d]	Technology used in COVID-19 vaccine[e]	Producing country of COVID-19 vaccine[f]
	Multivariate OR(95%CI)	Multivariate OR(95%CI)	Multivariate OR(95%CI)	Multivariate OR(95%CI)	Multivariate OR(95%CI)	Multivariate OR(95%CI)
Age						
18-29	1.00(reference.)	1.00(reference.)	1.00(reference.)	1.00(reference.)	1.00(reference.)	1.00(reference.)
30-39	1.13(0.60,2.12)	0.78(0.46,1.31)	0.75(0.38,1.47)	0.95(0.56,1.61)	0.61(0.24,1.45)	1.31(0.76,2.26)
40-49	0.44(0.20,0.92)*	0.45(0.26,0.77)*	1.10(0.52,2.35)	0.60(0.34,1.06)	0.69(0.26,1.71)	1.86(1.04,3.38)*
50-59	0.56(0.26,1.14)	0.43(0.25,0.73)*	1.09(0.51,2.36)	0.69(0.39,1.22)	0.41(0.12,1.13)	1.24(0.70,2.19)
60 and above	0.44(0.23,0.84)*	0.56(0.34,0.90)*	0.89(0.46,1.68)	0.74(0.45,1.21)	0.33(0.12,0.86)	1.28(0.78,2.11)
Gender						
Male	1.00(reference.)	1.00(reference.)	1.00(reference.)	1.00(reference.)	1.00(reference.)	1.00(reference.)
Female	0.99(0.63,1.54)	1.23(0.89,1.70)	1.30(0.83,2.03)	1.23(0.87,1.73)	1.02(0.53,1.96)	1.58(1.11,2.26)*
Highest education attainment						
Secondary school and below	1.00(reference.)	1.00(reference.)	1.00(reference.)	1.00(reference.)	1.00(reference.)	1.00(reference.)
Certificate/A-Level/Diploma	1.19(0.62,2.27)	1.32(0.78,2.22)	0.84(0.39,1.77)	0.75(0.43,1.27)	0.36(0.05,1.59)	1.08(0.61,1.92)
Bachelor degree	0.76(0.44,1.33)	0.64(0.41,0.97)*	0.70(0.37,1.26)	0.66(0.42,1.03)	1.22(0.51,3.28)	1.08(0.68,1.72)
Postgraduate degree	0.22(0.08,0.51)*	0.69(0.41,1.15)	0.80(0.38,1.66)	0.69(0.40,1.19)	2.26(0.89,6.27)	0.79(0.45,1.35)
Ever delayed acceptance or refuse vaccine despite availability of vaccine service						
No	1.00(reference.)	1.00(reference.)	1.00(reference.)	1.00(reference.)	1.00(reference.)	1.00(reference.)
Yes	1.73(0.88,3.22)	1.11(0.66,1.87)	1.15(0.59,2.49)	1.62(0.95,2.71)	3.35(1.53,6.96)*	0.85(0.50,1.47)

a: Only accept single dose vs Do not mind, b: Only accept 90% threshold vs Do not mind, c: Only accept minor adverse reactions vs Do not mind moderate adverse reactions, d: Only accept lesser than 12 months vs Do not mind moderate adverse reactions, e: Do not accept mRNA technology vs Do not know much about mRNA technology/Do not mind, f: Only accept a vaccine that is produced by specific countries vs Producing countries of a COVID-19 vaccine is not of my concern in vaccine choice

Acceptance and Preference for COVID-19 Vaccine in Japan

新型コロナウイルス感染症（COVID-19）ワクチン接種に関する考え方の研究

Section A

GENERAL INFORMATION

セッションＡ　一般情報

1	Age group<br>年齢（才）	• 18-29<br>• 30-39<br>• 40-49<br>• 50-59<br>• 60-69<br>• 70-79<br>• 80-89<br>• 90-99<br>• 100 ～
2	Gender<br>性別	• Male 男<br>• Female 女
3	Highest education attainment<br>学歴	• Primary school/ 小学校及び未満<br>• Secondary school (high school) or below<br>高校及び未満<br>• Diploma or equivalency certificate<br>短大・専門学校<br>• Bachelor's degree 大学<br>• Postgraduate degree 大学院
4	Countries 居住国	• Japan　日本
5	Have you ever delay acceptance or refuse of vaccines despite availability of vaccine service?<br>過去にワクチンの接種を拒否または期間内に接種しなかったことがありますか？	• Yes はい<br>• No いいえ

COVID-19 vaccine acceptance
新型コロナウイルスワクチンの接種に関して

1	Would you accept the COVID-19 vaccine if it is recommended by the government in your country and the COVID-19 vaccination service is available?<br>政府が推奨する新型コロナウイルスワクチンの接種ができる場合、その接種を希望しますか？	• Extremely likely　大いにしたい<br>• Likely　したい<br>• Unlikely　あまりしたくない<br>• Extremely unlikely　絶対にしない

Attitudes towards COVID-19 vaccine characteristics
新型コロナウイルスワクチン接種に関する考え

1	The following are regarding the required **doses** of COVID-19<br>新型コロナウイルスワクチンの接種回数に関して：<br>[　　] I will only accept the vaccine if just a single vaccine dose is required<br>１回投与で済むワクチンなら接種する<br>[　　] I do not mind if a booster dose is required following a primary vaccination<br>２回接種が必要なワクチンでも構わない
2	The following are regarding the **effectiveness** threshold of COVID-19 vaccine<br>新型コロナウイルスワクチンの有効性に関して：<br>[　　] I will only accept a vaccine that reported nearly 90% effective or above in preventing COVID-19<br>有効性 90%以上のものだけを接種する<br>[　　] I do not mind receiving a vaccine that reported below 90% effective in preventing COVID-19 vaccine<br>有効性 90%以下のものでも構わない
3	The following are regarding **side-effects** of COVID-19 vaccine<br>新型コロナウイルスワクチンの副作用に関して：：

	[ ] I will only accept a vaccine that has minor side effects such as soreness, swelling at the injection site that do not disrupt daily life.<br>注射部位の痛みや腫れなど、日常生活に支障のない軽微な副作用のあるワクチンならば受ける。<br>[ ] I do not mind if the vaccine has moderate side effects such as fever, headaches that disrupt daily life<br>発熱や頭痛など日常生活に支障をきたす中程度の副作用があっても構わない
4	The following are regarding the **duration of protection** of COVID-19 vaccine<br>新型コロナウイルスワクチンの有効期間に関して：<br><br>[ ] I will only accept a vaccine with duration protection no shorter than 12 months<br>有効期間が 12 か月以上のものしか受けない<br>[ ] I do not mind if the duration of protection of vaccine between 6 to 12 months<br>有効期間が 6～12 か月でも受ける
5	**Technology used in** COVID-19 vaccine production<br>新型コロナウイルスワクチンの生産技術に関して：<br>[ ] I will NOT accept a COVID-19 developed using mRNA technology<br>mRNA 技術を使ったワクチンは受けたくない<br>[ ] I do not mind receiving an mRNA COVID-19 vaccine<br>mRNA 技術を使ったワクチンでも構わない<br>[ ] I do not know much about mRNA technology<br>mRNA 技術に関する知識があまりない
6	The following are regarding the **producing country** of COVID-19 vaccine<br>ワクチンの生産国に関して<br>[ ] I will only accept a vaccine that is produced by specific countries<br>特定の国で製造されたワクチンだけを受けたい<br>[ ] The producing countries of a COVID-19 vaccine is not of my concern in vaccine choice.<br>ワクチンを選ぶときに、生産国にはこだわらない。

Factor influencing COVID-19 vaccine choice
新型コロナウイルスワクチンの選択に関する要因

1	Which of the following is the **FIRST foremost important** factor influencing your choice of COVID-19 vaccine.<br>貴方にとって、新型コロナウイルスワクチンを選択する時、最も重要視するのは何ですか？<br>[ ] Number of doses 接種する回数<br>[ ] Effectiveness threshold 有効性<br>[ ] Side-effects 副作用<br>[ ] Duration of protection 有効期間<br>[ ] Producing countries 生産国<br>[ ] Total cost of vaccination (including booster if required) 費用
2	Which of the following is the **SECOND foremost important** factor influencing your choice of COVID-19 vaccine.<br>貴方にとって、新型コロナウイルスワクチンを選択する時、2番目に重要視するのは何ですか？<br>[ ] Number of doses 接種する回数<br>[ ] Effectiveness threshold 有効性<br>[ ] Side-effects 副作用<br>[ ] Duration of protection 有効期間<br>[ ] Producing countries 生産国<br>[ ] Total cost of vaccination (including booster if required) 費用
